# Tackling Antimicrobial Resistance in the Shadow of COVID-19

**DOI:** 10.1128/mBio.00473-21

**Published:** 2021-07-20

**Authors:** Ami Patel

**Affiliations:** a Vaccine and Immunotherapy Center, The Wistar Institute, Philadelphia, Pennsylvania, USA

**Keywords:** AMR, DNA-encoded antibodies, antimicrobial resistance, bacteria, gene-encoded antibodies, personalized medicine

## Abstract

The coronavirus disease 2019 (COVID-19) pandemic is a challenge for ongoing efforts to combat antimicrobial-resistant (AMR) bacterial infections. As we learn more about COVID-19 disease and drug stewardship evolves, there is likely to be a lasting impact of increased use of antimicrobial agents and antibiotics, as well as a lack of consistent access to health care across many populations. Sexually transmitted infections have been underreported during the pandemic and are often caused by some of the most drug-resistant pathogens. In their recent article in *mBio*, Parzych et al. (E. M. Parzych, S. Gulati, B. Zheng, M. A. Bah, et al., mBio 12:e00242-21, 2021, https://doi.org/10.1128/mBio.00242-21) focus on protection against Neisseria gonorrhoeae infection via *in vivo* delivery of an antigonococcal DNA-encoded antibody that has been modified for increased complement activation. Nucleic acid approaches are highly adaptable and could be tremendously beneficial for personalized strategies to combat AMR pathogens.

## COMMENTARY

Antimicrobial resistance (AMR) is one of the most serious threats to public health. There are approximately 700,000 deaths/year globally due to AMR, and this number is estimated to balloon to 10 million AMR deaths/year by the year 2050 unless urgent measures are taken to combat the spread of drug-resistant organisms. Global strategies to combat AMR and prevent the emergence of new drug-resistant organisms are becoming further complicated in the era of coronavirus disease 2019 (COVID-19).

The COVID-19 pandemic is placing enormous burdens on global health care and presents many difficulties for appropriate antimicrobial use and stewardship. The direct impact of COVID-19 on AMR and emergence of new multidrug-resistant organisms is yet to be fully understood. There is increased antimicrobial use in hospitals and community settings, especially early during the pandemic when less information was available. Up to 70% of those treated for COVID may have received a concurrent course of antibiotics to prevent bacterial infections as well as to treat infections (1, 2). Secondary infections with some of the most multidrug-resistant (MDR) bacteria such as *Enterobacterales* spp., MDR Pseudomonas aeruginosa, and Acinetobacter baumannii have been reported (3–5). COVID-19 has led to increased hospitalization, including mechanical ventilation and extended duration of stay, both high risks for acquiring AMR infections. While this has been less of a problem for the U.S. and European countries, a recent study from India shows that coinfection rates are higher and leading to increased mortality (6). Additionally, for many people worldwide, there has been delayed or lack of access to routine health care and general reticence for in-person visits for fear of COVID-19 infection. Bacterial coinfections and secondary infection following severe acute respiratory syndrome coronavirus 2 (SARS-CoV-2) infection/COVID-19 are important considerations for understanding shifts in AMR patterns.

The COVID-19 pandemic has had an impact on diagnosis and reporting of sexually transmitted infections (STIs), with potentially serious implications for development of AMR infections. The National Coalition of STD Directors reported on the disruption of COVID-19 to intervention services for chlamydia, gonorrhea, and syphilis infections (7). Multiple centers across the United States are reporting an increase in all three bacterial STIs during the pandemic. While all are treatable, the pandemic may exacerbate the emergence of multidrug-resistant strains. It is estimated that around 87 million people are infected by gonorrhea each year (8) and super-drug-resistant gonorrhea infections that are refractory to first-line antibiotics have been reported in multiple regions including South East Asia, Japan, Spain, United Kingdom, and Australia (reviewed in reference 9). In the United States, the CDC reports an alarming 63% increase in gonococcal infections since 2014 (10). In their recent article in *mBio*, Parzych et al. (11) describe *in vivo* DNA delivery of an Fc engineered antigonococcal antibody 2C7 that targets the surface lipooligosaccharide (LOS) protein as a preventative strategy against Neisseria gonorrhoeae infection.

Overcoming infections with AMR bacteria has been an ongoing challenge since shortly after the introduction of antibiotics. While there are several classes of broad-spectrum antibiotics for treating infections with Gram-positive and Gram-negative bacteria, many pathogens are rapidly evolving or acquiring resistance to first-line treatments and will respond only to last-resort antibiotics. There are significant efforts to discover new antibiotic classes and develop derivatives and multidrug combinations. Recent high-throughput small molecule screens for compounds that target bacterium-specific pathways are promising (12, 13); however, additional strategies such as vaccination and biologics that can be codelivered with the current standard of care have the potential to be beneficial as part of the arsenal to combat AMR infections.

Highly potent, pathogen-specific monoclonal antibodies (MAbs) are increasingly attractive biologics against AMR pathogens. The FDA has approved MAbs targeting inhalational anthrax (raxibacumab and obiltoxamab) and anti-Clostridium difficile toxin B (bezlotoxumab) in conjunction with the standard of care. MAbs against other bacterial pathogens in human phase trials include Staphylococcus aureus alpha hemolysin (Aridis and AstraZeneca) and Pseudomonas aeruginosa (reviewed in reference 14). Gulati et al. previously described antibody 2C7 targeting the N. gonorrhoeae LOS, demonstrating efficacy in mice following recombinant delivery of a modified 2C7 MAb that enhanced complement activation leading to improved therapeutic bacterial clearance (15). However, high doses of recombinant antibody are required for therapeutic clearance. Several advances in half-life extension are leading to longer antibody trough levels in sera; however, significant hurdles remain for routine uptake of MAb delivery for AMR prevention including dose, costs, and biologic stability. Gene-delivered antibodies using nucleic acid (DNA or mRNA) or viral vector (adeno-associated virus [AAV]) platforms is a rapidly advancing field that has tremendous potential to expand the delivery of protective MAbs against AMR pathogens (reviewed in reference 16).

In their recent article in *mBio*, Parzych et al. directly injected mice with plasmid DNA encoding the immunoglobulin heavy and light chains of MAb 2C7_WT and Fc variants designed to activate complement via IgG hexamerization (2C7_E345K and 2C7_E430G) (11). The DNA-encoded monoclonal antibody (DMAb) version of 2C7 was expressed for several months and afforded preventative protection in mice against mucosal N. gonorrhoeae challenge at early time points postadministration, as well as demonstrated protection against rechallenge several months later. The authors show that low doses of the 2C7_E430G DMAb were protective at preventing mucosal N. gonorrhoeae infection. This study highlights that an antibody expressed from muscle following *in vivo* delivery can provide protection at a distal mucosal site (vaginal challenge). Additionally, they show long-term protection against a difficult-to-treat pathogen. As the incidence of gonorrhea and other STIs increase, approaches such as DMAb delivery and other gene-delivered approaches could become key to reducing the emergence of AMR and preventing new infections in at-risk populations. *In vivo* delivery of biologics with increased potency such as 2c7_E430G DMAb as described by Parzych et al. could have an important impact on the course of AMR infection.

**FIG 1:**
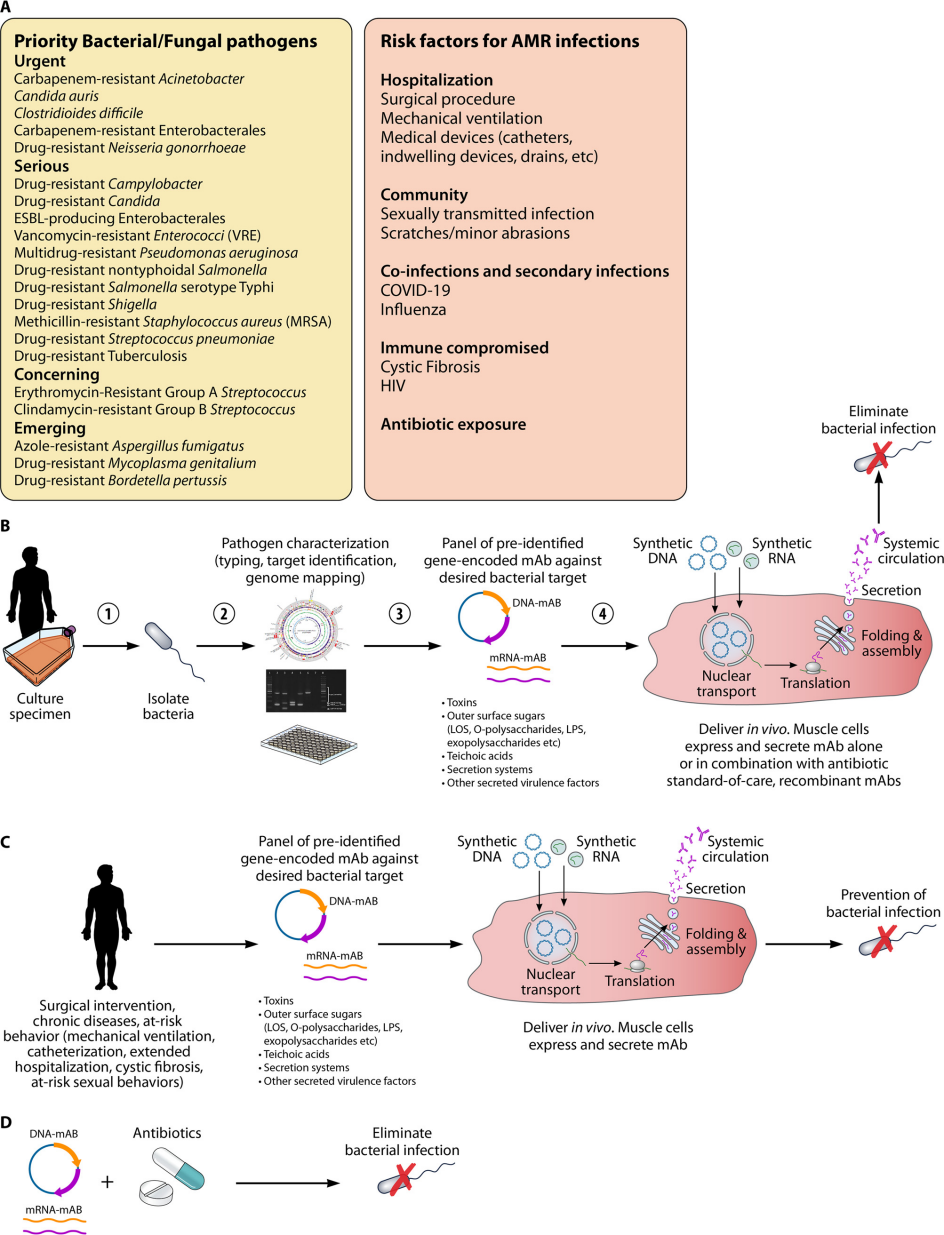
Personalizing delivery of gene-encoded antibodies against antimicrobial-resistant pathogens. (A) Overview of priority bacterial and fungal pathogens and risk factors of concern for AMR (CDC and WHO). (B) Personalizing therapeutic protection against resistant bacteria. Following specimen collection, bacteria are cultured and typed for identification and characterization. A preidentified panel of gene-encoded antibodies can be delivered to the patient as an off-the-shelf intervention. Gene-encoded MAbs enter cells and express and secrete functional antibodies that lead to pathogen elimination. (C) Personalizing preventative protection against resistant bacteria. The preidentified panel of gene-encoded antibodies is delivered in advance of a surgical intervention, chronic disease, or at-risk behavior. The gene-encoded MAbs enter cells and express and secrete functional antibodies that prevent infection. (D) Gene-encoded MAbs may be delivered alone or in conjunction with the antibiotic standard of care or recombinant antibodies.

Collectively, the medical field is moving toward different aspects of personalized medicine to help tackle challenges like cancer and genetic diseases. Although infectious diseases are a part of daily life, their impact is often underappreciated, and AMR strategies have yet to embrace more personalized approaches (Fig. 1). It is conceivable that gene-encoded antibodies could enable delivery of pathogen-specific protective MAbs to high-risk populations, for example people undergoing surgical procedures, long-term hospitalization, mechanical ventilation, and medical implants. Those who are immunocompromised and individuals with cystic fibrosis who are at increased risk for acquiring bacterial and AMR infections would greatly benefit from a personalized approach. Furthermore, prevention campaigns in at-risk populations for bacterial STIs similar in concept to HIV preexposure prophylaxis (PReP) could have a direct impact on slowing the emergence of AMR organisms. As we begin to emerge from the large shadow cast by COVID-19, combination delivery of gene-encoded antibodies and other personalized standard-of-care regimens have great potential to reduce the overall global burden of AMR.
